# Supplementary figures and images for: Merkel cell polyomavirus protein ALTO modulates TBK1 activity to support persistent infection
Source: PLoS Pathog. 2024 Jul 29;20(7):e1012170. doi: 10.1371/journal.ppat.1012170 (PMC11285941; doi:10.1371/journal.ppat.1012170)

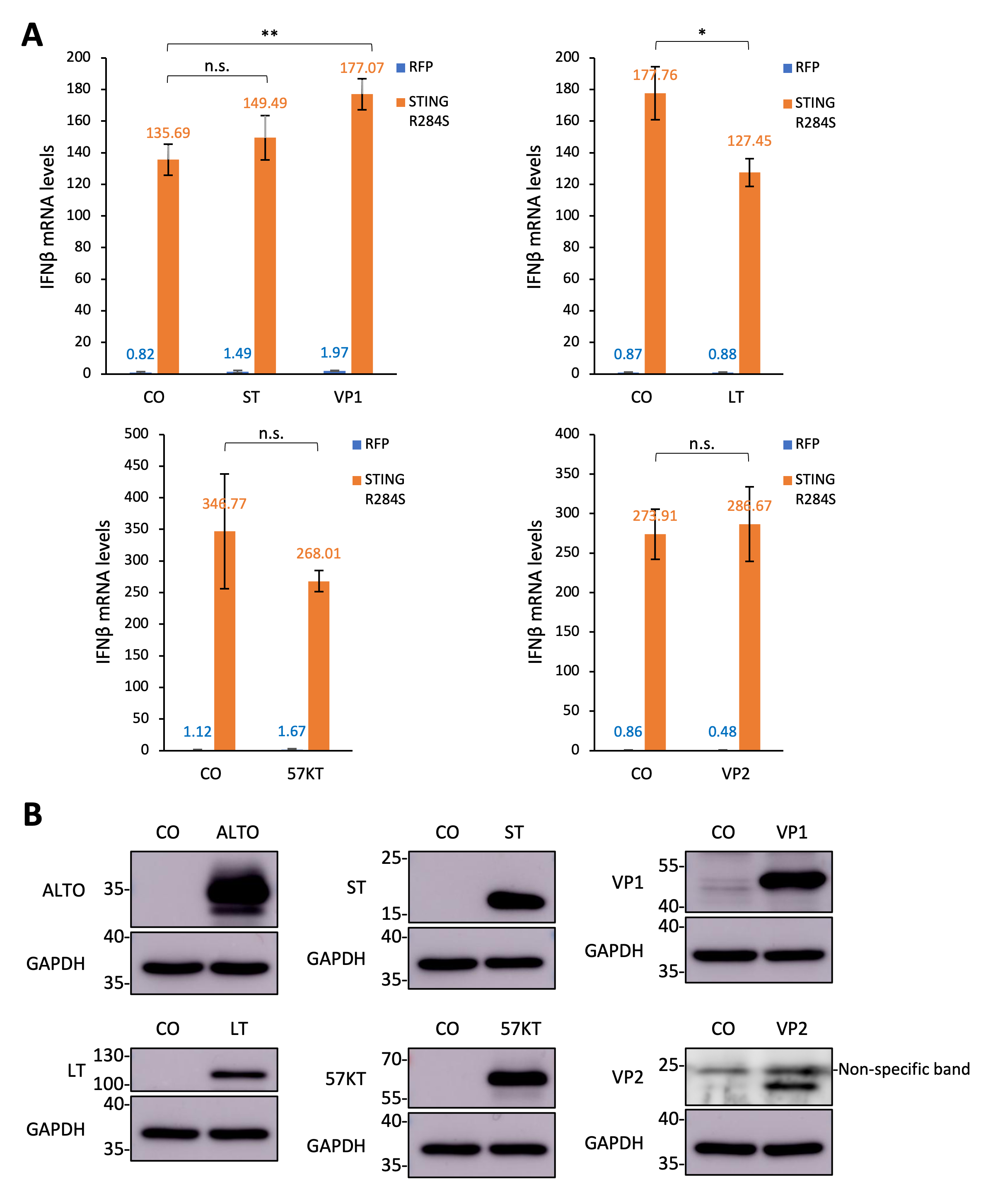

Supplement: S1 Fig — A. HEK293 cells were transfected with plasmids carrying indicated viral proteins (or empty vector controls) and STINGR284S (or RFP control). At 2 days post-transfection, IFNβ mRNA levels were measured using RT-qPCR. One of the values for transfection with vector and RFP was set as 1. Error bars indicate the standard deviation from three independent samples. **p<0.01; *p<0.05; n.s. = not significant. B. HEK293 cells were transfected with plasmids encoding the indicated viral proteins or their respective empty vector controls. Whole cell lysates were then blotted with the indicated antibodies to confirm the expression of the viral proteins. (TIF) [file ppat.1012170.s001.tif]

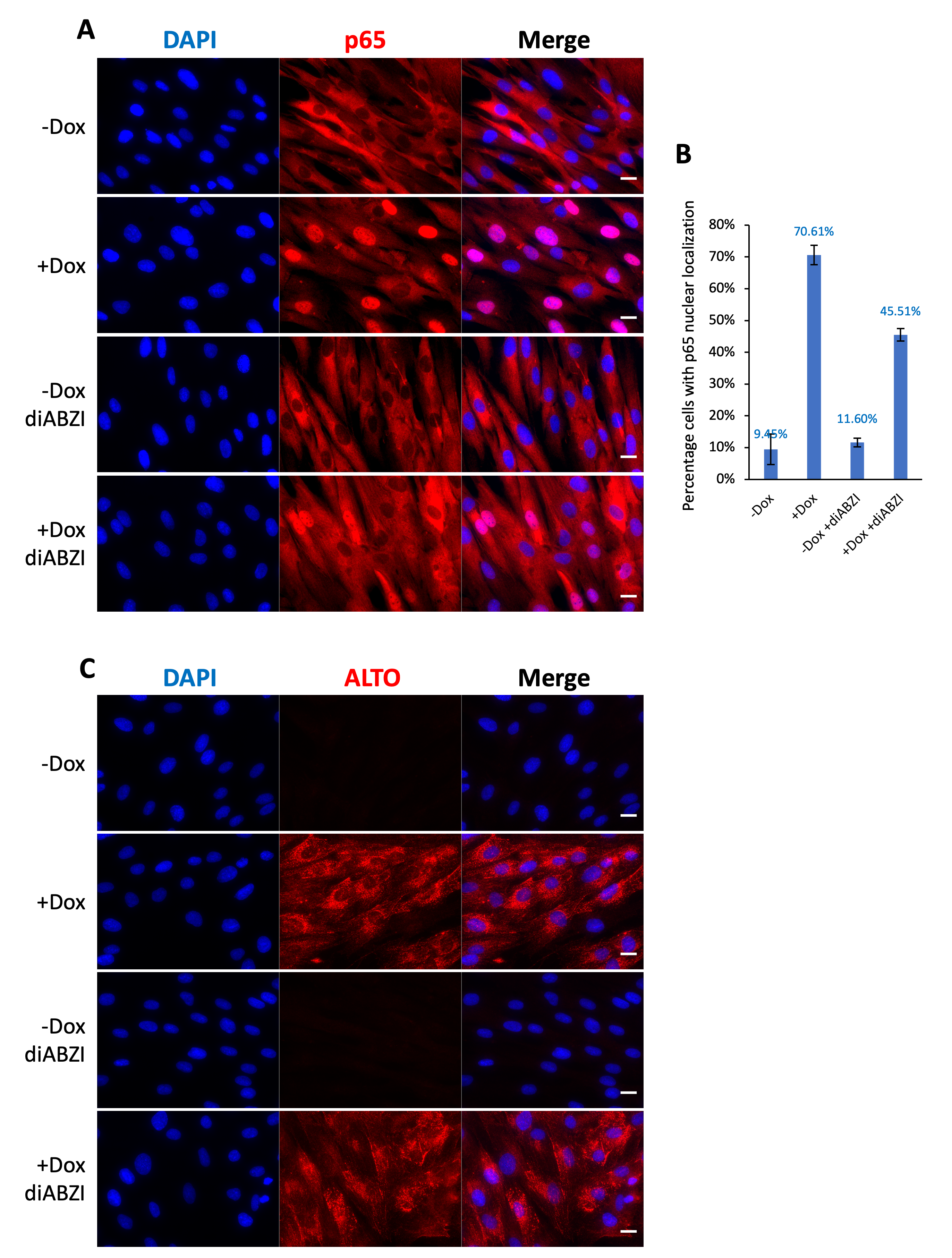

Supplement: S2 Fig — A. HDF-inALTO or -inRFP cells were mock-treated or induced with Dox for 8 hours before being stimulated with diABZI (or DMSO) for an additional 16 hours. Cells were fixed and immunostained for p65, and counterstained with DAPI. Scale bar, 20 μm. B. Quantification of the percentages of cells with p65 nuclear localization for cells treated as in A. Bars indicate the mean; error bars represent standard deviation from 3 replicates. C. Cells treated as in A were fixed and immunostained for ALTO and counterstained with DAPI. Scale bar, 20 μm. (TIF) [file ppat.1012170.s002.tif]

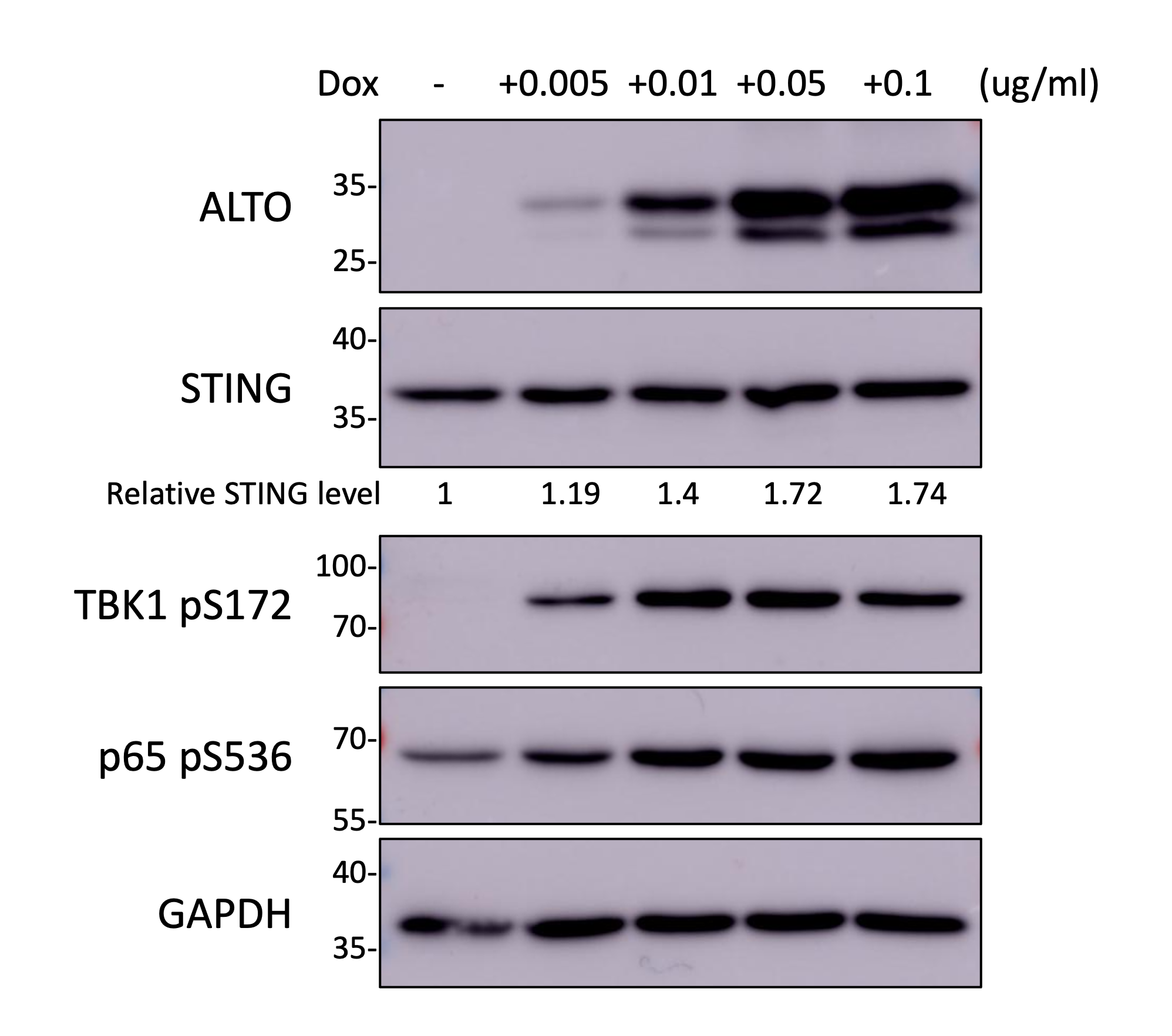

Supplement: S3 Fig — HDF-inALTO cells were mock-treated or induced with the indicated doses of Dox for 42 hours. Whole cell lysates were resolved by SDS/PAGE and immunoblotted with the indicated antibodies. Relative abundance of STING was calculated in ImageJ by setting the value of the band in the uninduced cells to 1. (TIF) [file ppat.1012170.s003.tif]

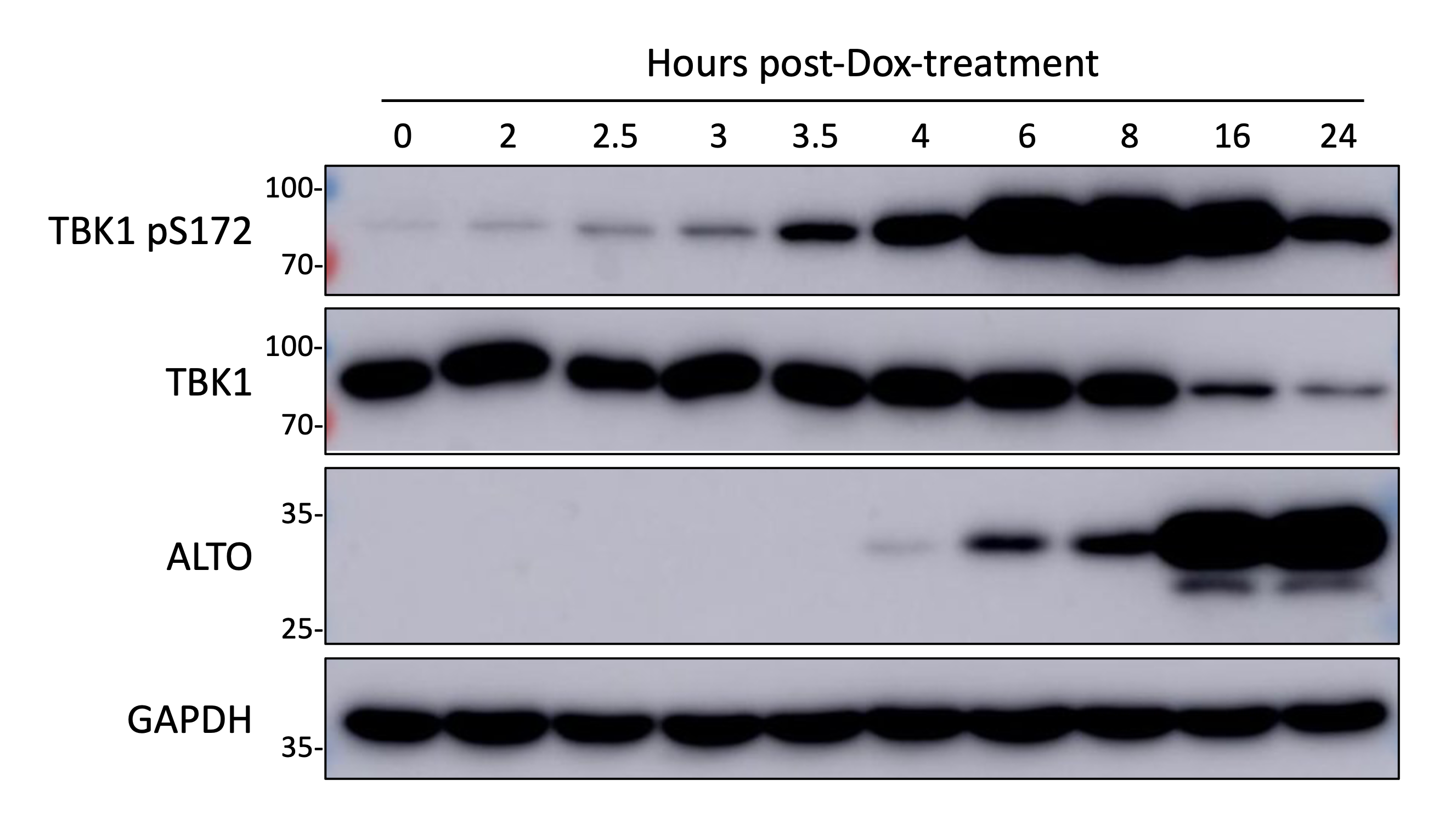

Supplement: S4 Fig — HDF-inALTO cells were mock-treated or induced with Dox for the indicated times. Whole cell lysates were resolved by SDS/PAGE and immunoblotted with the indicated antibodies. (TIF) [file ppat.1012170.s004.tif]

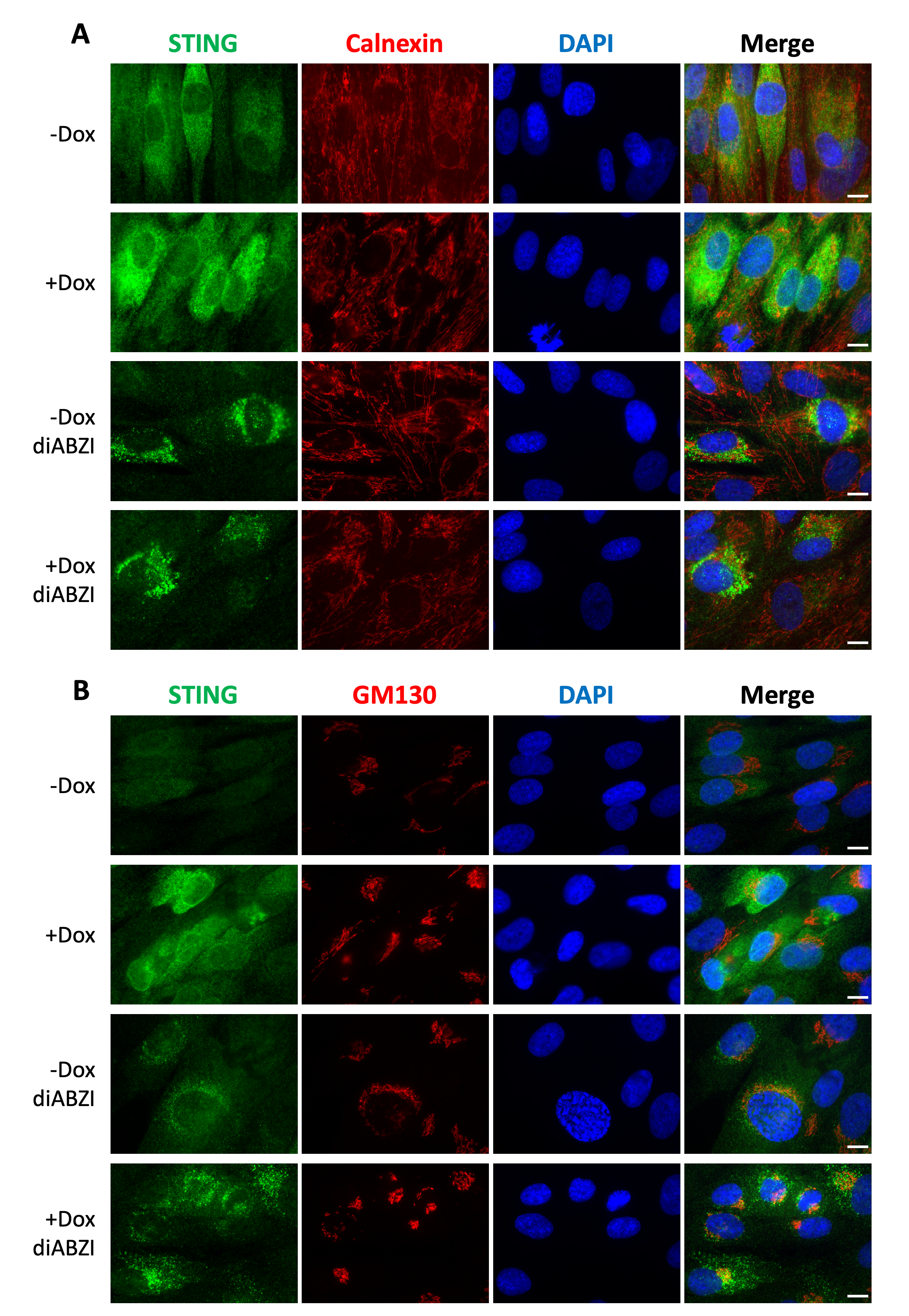

Supplement: S5 Fig — A. HDF-inALTO cells were mock-treated or induced with Dox for 24 hours before being stimulated with diABZI (or DMSO) for another 16 hours. Cells were fixed and immunostained for STING and Calnexin, and counterstained with DAPI. B. Cells treated as in A were fixed and immunostained for STING and GM130, and counterstained with DAPI. Scale bar, 10 μm. (TIF) [file ppat.1012170.s005.tif]

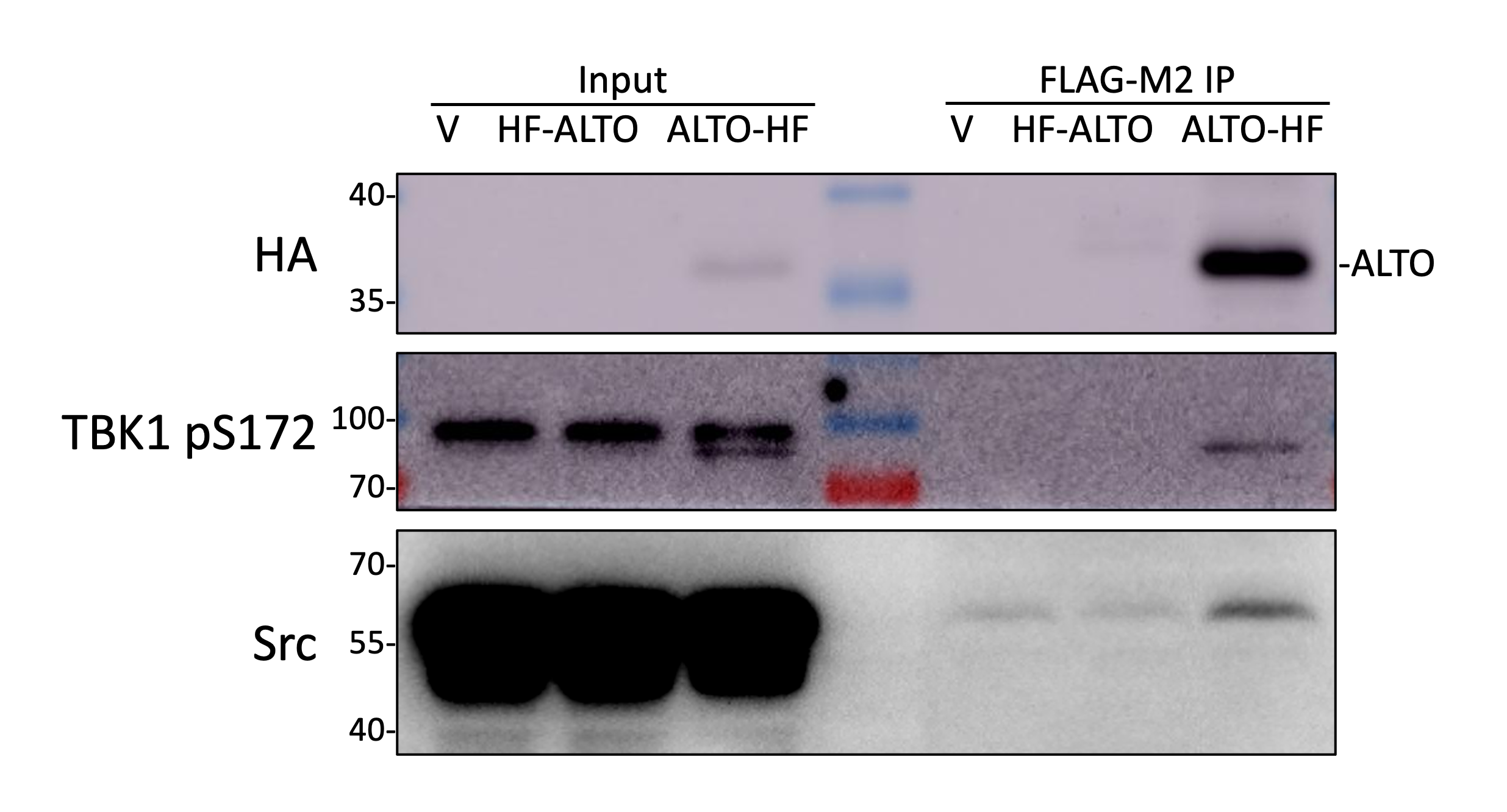

Supplement: S6 Fig — HEK293 cells were transfected with empty vector control or vector containing N- or C-terminal HA- and FLAG-tagged ALTO. At 40-hour post-transfection, whole cell lysates were incubated with Anti-FLAG M2 affinity gel. Immunoprecipitants were resolved by SDS/PAGE and immunoblotted with the indicated antibodies. (TIF) [file ppat.1012170.s006.tif]

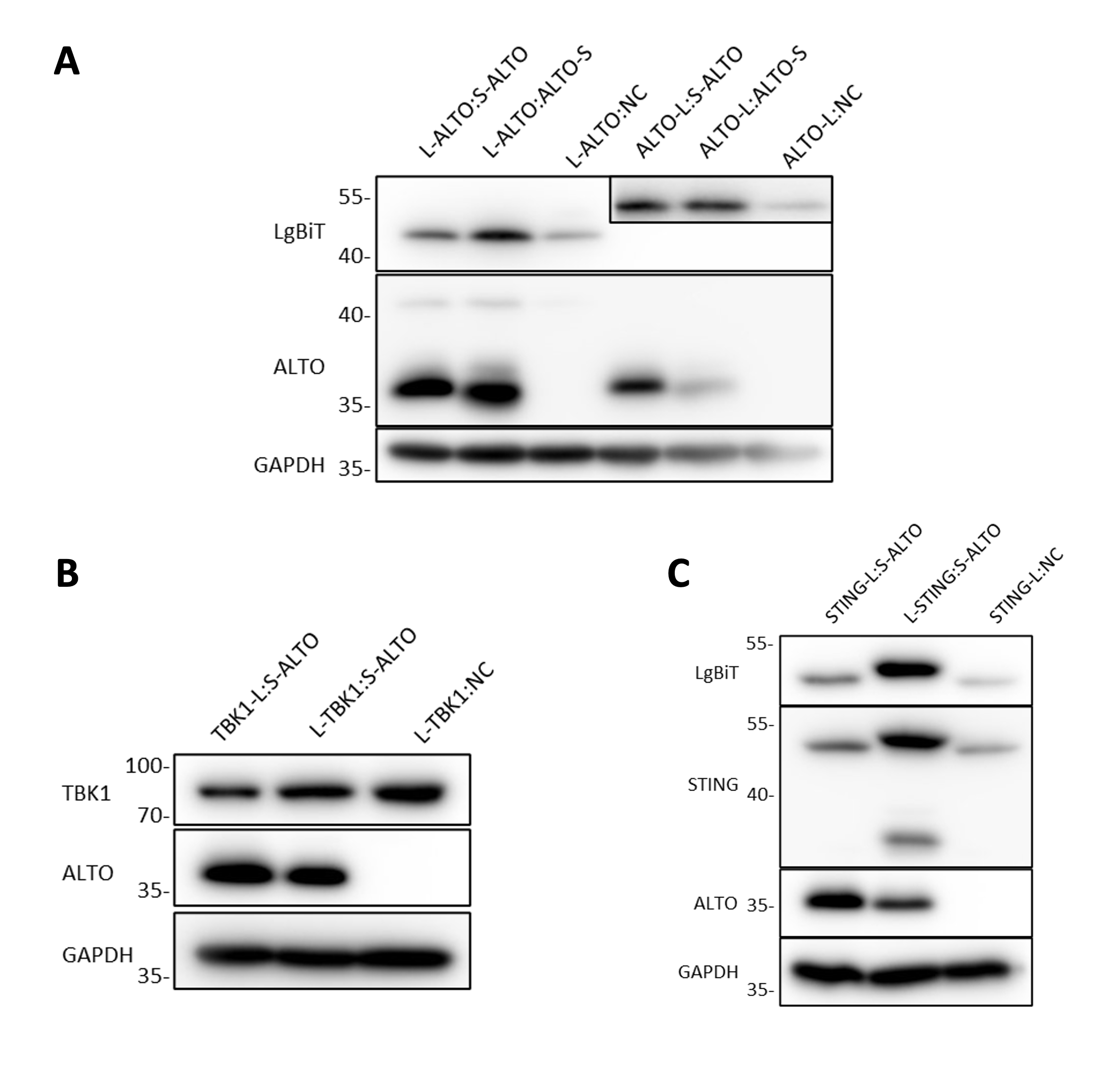

Supplement: S7 Fig — A. HEK293 cells were transfected with pairs of constructs carrying NanoBiT- (Large BiT or SmBiT) fused ALTO as indicated. At 24 hours post-transfection, whole cell lysates were collected and resolved by SDS/PAGE and immunoblotted with the indicated antibodies. Inset: To enhance visibility of the faint ALTO-LgBiT bands in the last three lanes, the LgBiT-blotted membrane was re-imaged with the LgBiT-ALTO bands in the first three lanes covered and the exposure time increased. B. HEK293 cells were transfected with pairs of constructs carrying NanoBiT- (Large BiT or SmBiT) fused ALTO and TBK1 as indicated. At 20 hours post-transfection, whole cell lysates were collected and resolved by SDS/PAGE and immunoblotted with the indicated antibodies. C. HEK293 cells were transfected with pairs of constructs carrying NanoBiT- (Large BiT or SmBiT) fused ALTO and STING as indicated. At 24 hours post-transfection, whole cell lysates were collected and resolved by SDS/PAGE and immunoblotted with the indicated antibodies. (TIF) [file ppat.1012170.s007.tif]

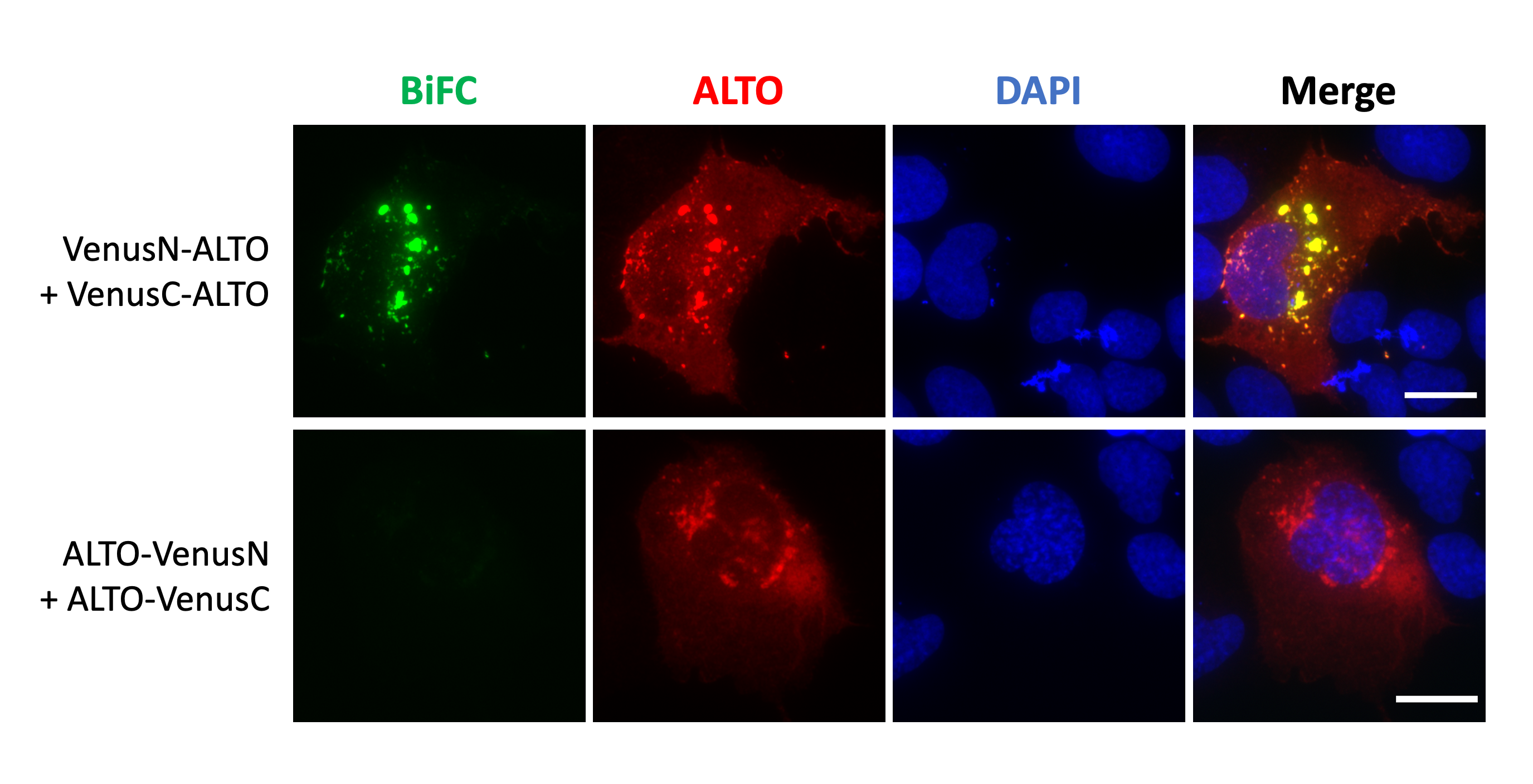

Supplement: S8 Fig — U2OS cells were transfected with pairs of plasmids carrying Venus (N-terminal half or C-terminal half) fused ALTO constructs. At 48-hour post-transfection, cells were fixed and immunostained for ALTO, and counterstained with DAPI. Scale bar, 20 μm. (TIF) [file ppat.1012170.s008.tif]

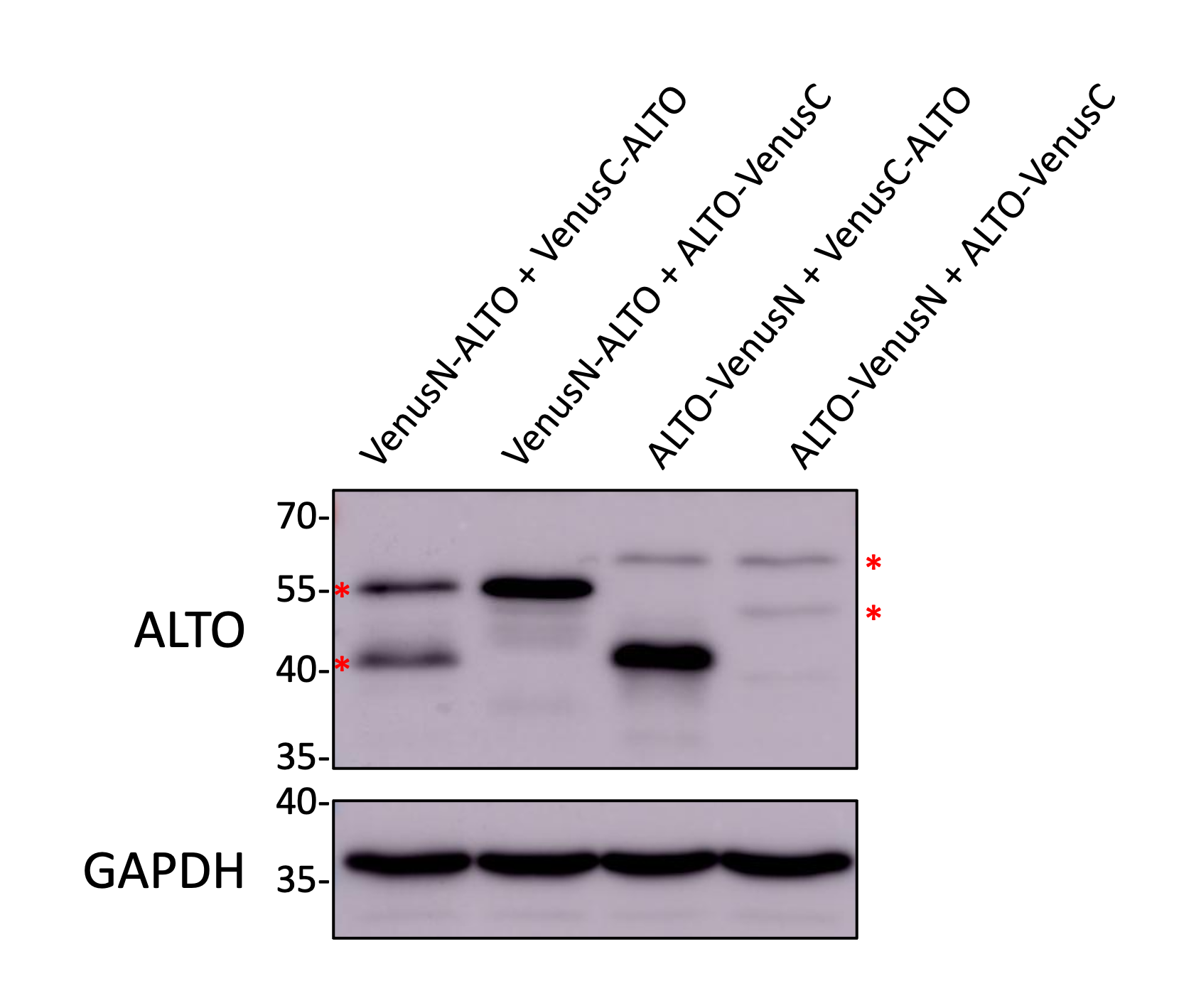

Supplement: S9 Fig — U2OS cells were transfected with pairs of plasmids carrying Venus (N-terminal half or C-terminal half) fused ALTO constructs as indicated. At 48-hour post-transfection, whole cell lysates were resolved by SDS/PAGE and immunoblotted with the indicated antibodies. Red asterisks indicate bands that reflect the position of full-length ALTO protein, which was variably shifted due to tagging. (TIF) [file ppat.1012170.s009.tif]

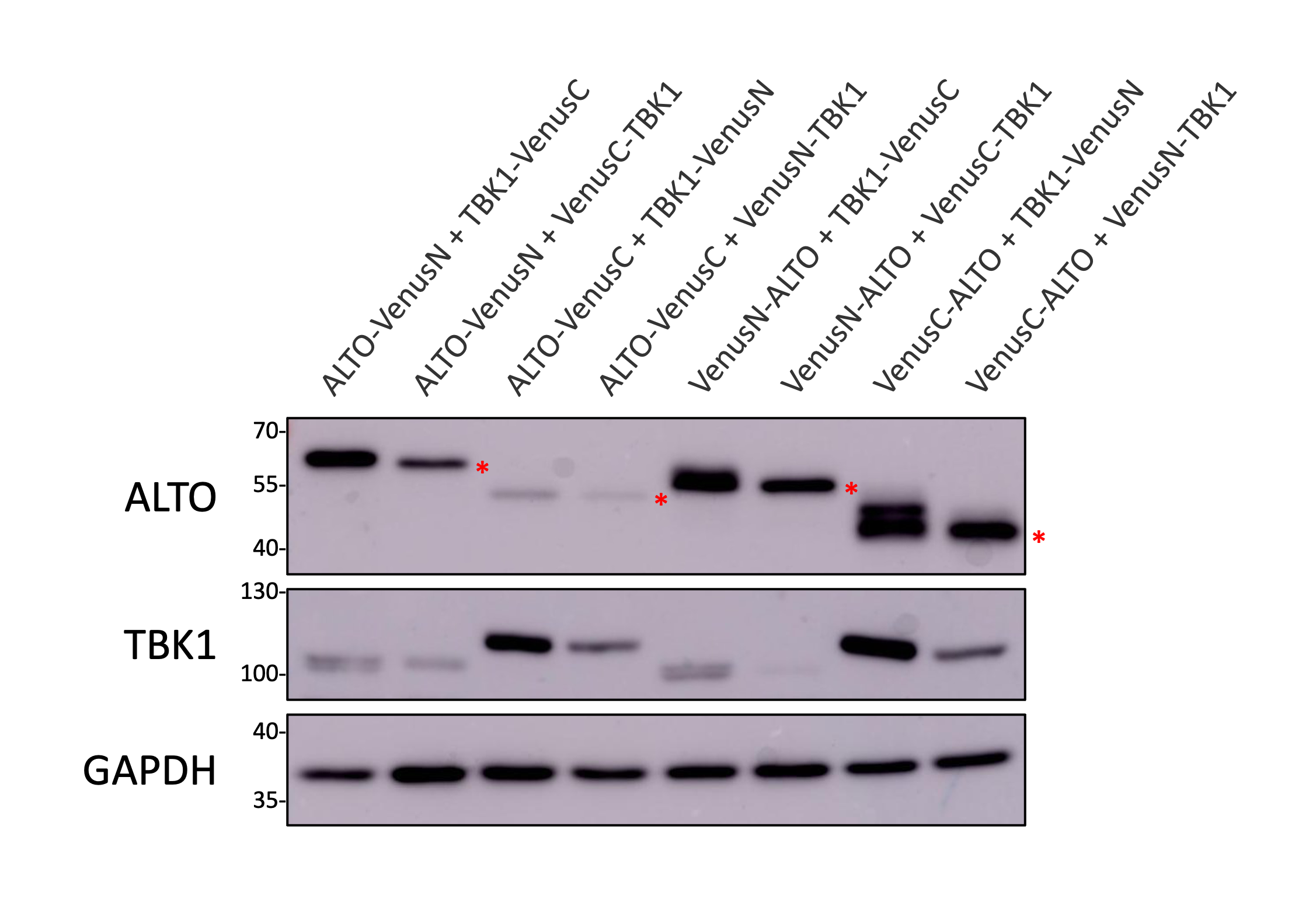

Supplement: S10 Fig — U2OS cells were transfected with pairs of plasmids carrying Venus (N-terminal half or C-terminal half) fused ALTO and TBK1. At 24-hour post-transfection, whole cell lysates were resolved by SDS/PAGE and immunoblotted with the indicated antibodies. Red asterisks indicate bands that reflect the position of full-length ALTO protein, which was variably shifted due to tagging. (TIF) [file ppat.1012170.s010.tif]

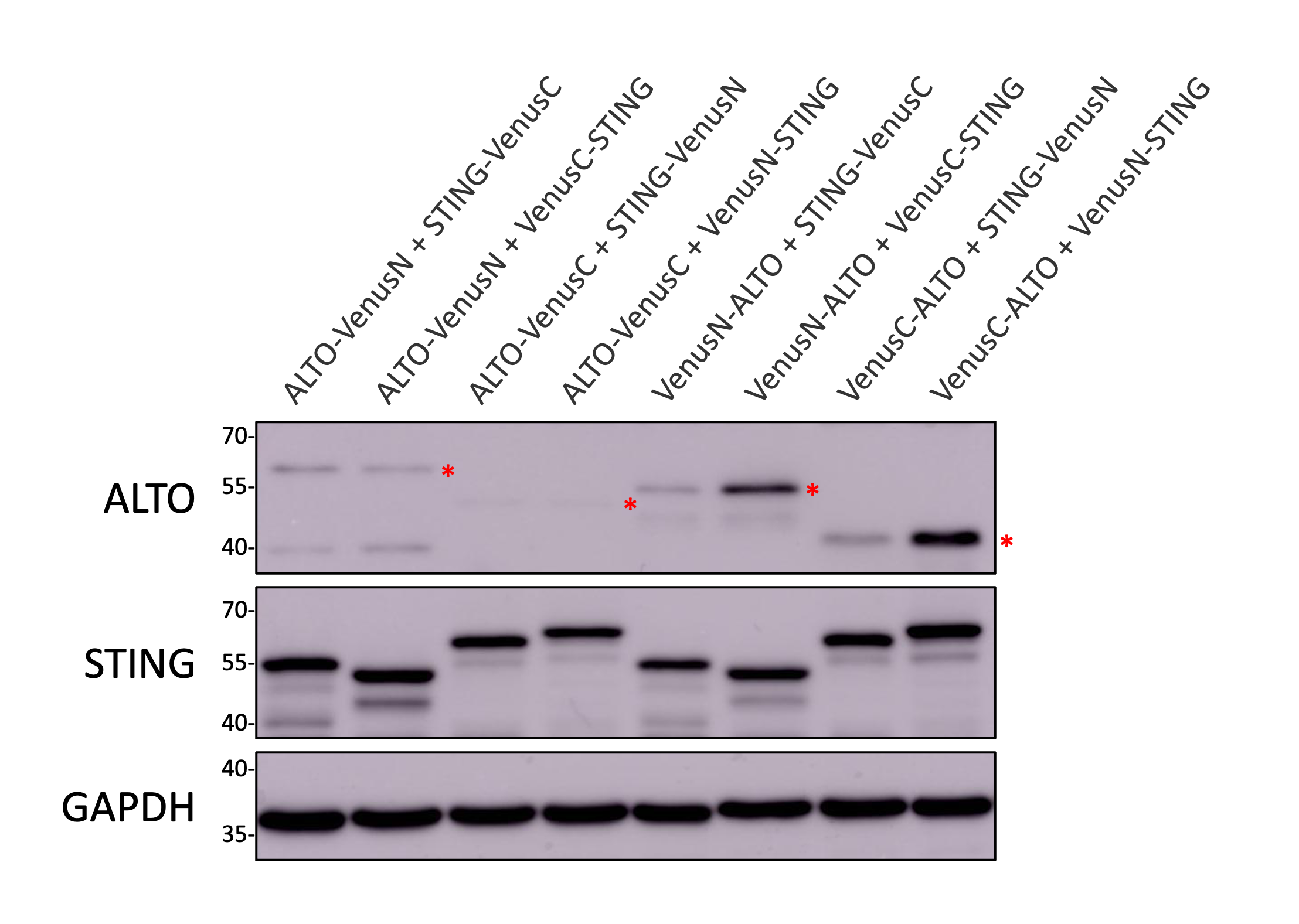

Supplement: S11 Fig — U2OS cells were transfected with pairs of plasmids carrying Venus (N-terminal half or C-terminal half)-fused ALTO and STING. At 24-hour post-transfection, whole cell lysates were resolved by SDS/PAGE and immunoblotted with the indicated antibodies. Red asterisks indicate bands that reflect the position of full-length ALTO protein, which was variably shifted due to tagging. (TIF) [file ppat.1012170.s011.tif]

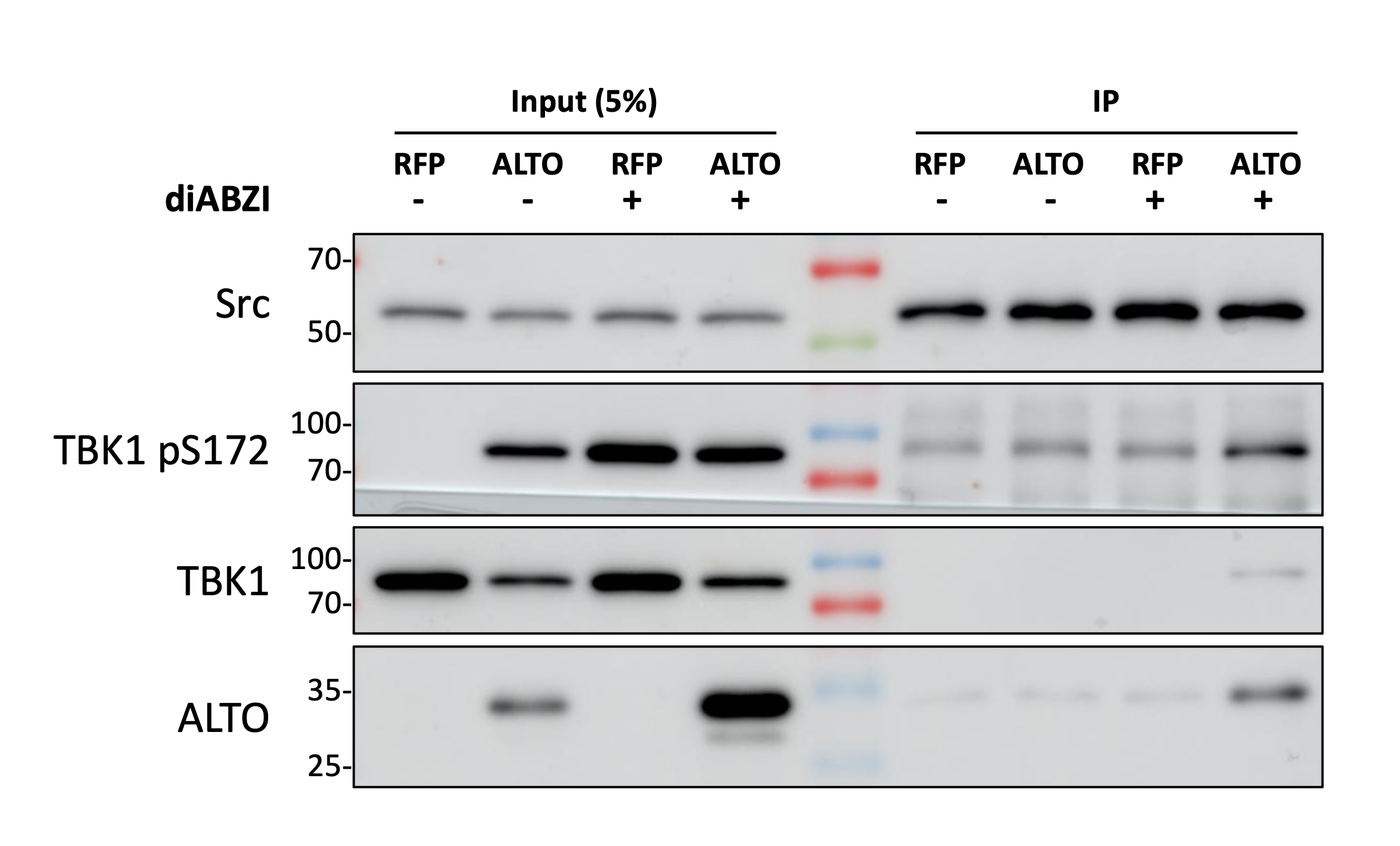

Supplement: S12 Fig — Normal HDF-inALTO or -inRFP cells were mock-treated or induced with Dox for 10.5 hours, then stimulated with diABZI (or DMSO control) for 1.5 hours. Whole cell lysates were incubated with rabbit anti-Src antibody, then immunoprecipitated with Protein G agarose. Immunoprecipitants were resolved by SDS/PAGE and immunoblotted with the indicated antibodies. (TIF) [file ppat.1012170.s012.tif]

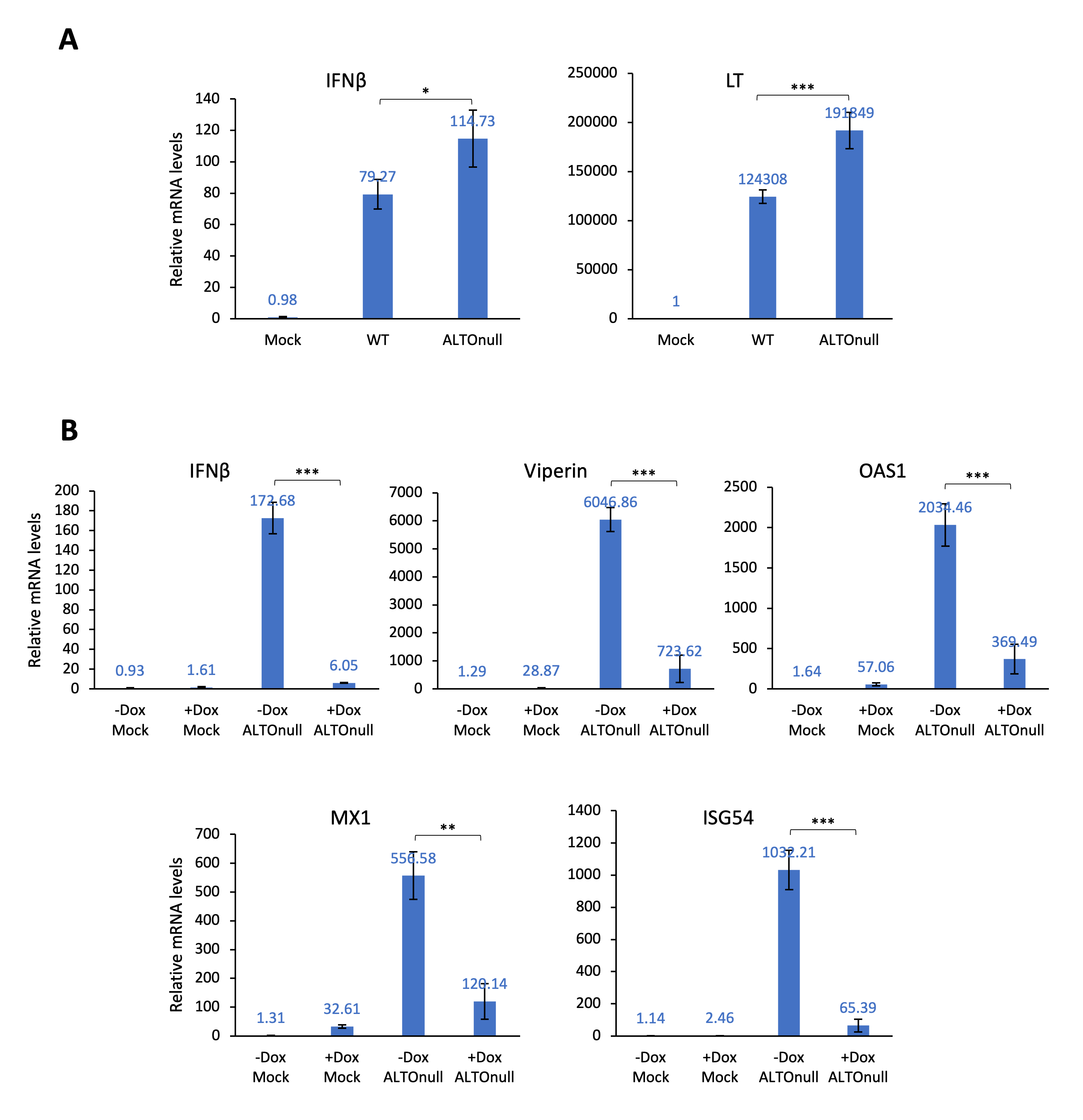

Supplement: S13 Fig — A. Normal HDFs were mock-infected or infected with WT or ALTOnull MCPyV. The cells were harvested at 5 days post-infection, and mRNA levels were quantified by RT-qPCR. The value for mock-infected HDFs was set to 1. Bars indicate the mean; error bars represent the standard deviation from 3 replicates. B. HDF-inALTO cells were mock-infected or infected with ALTOnull MCPyV. Cells were mock-treated or induced with Dox at 2- and 4-days post-infection. Cells were harvested at 5 days post-infection, and mRNA levels were quantified by RT-qPCR. The value for mock-infected and mock-treated HDFs was set to 1. Bars indicate the mean; error bars represent the standard deviation from 3 replicates. ***p<0.001; **p<0.01; *p<0.05. (TIF) [file ppat.1012170.s013.tif]

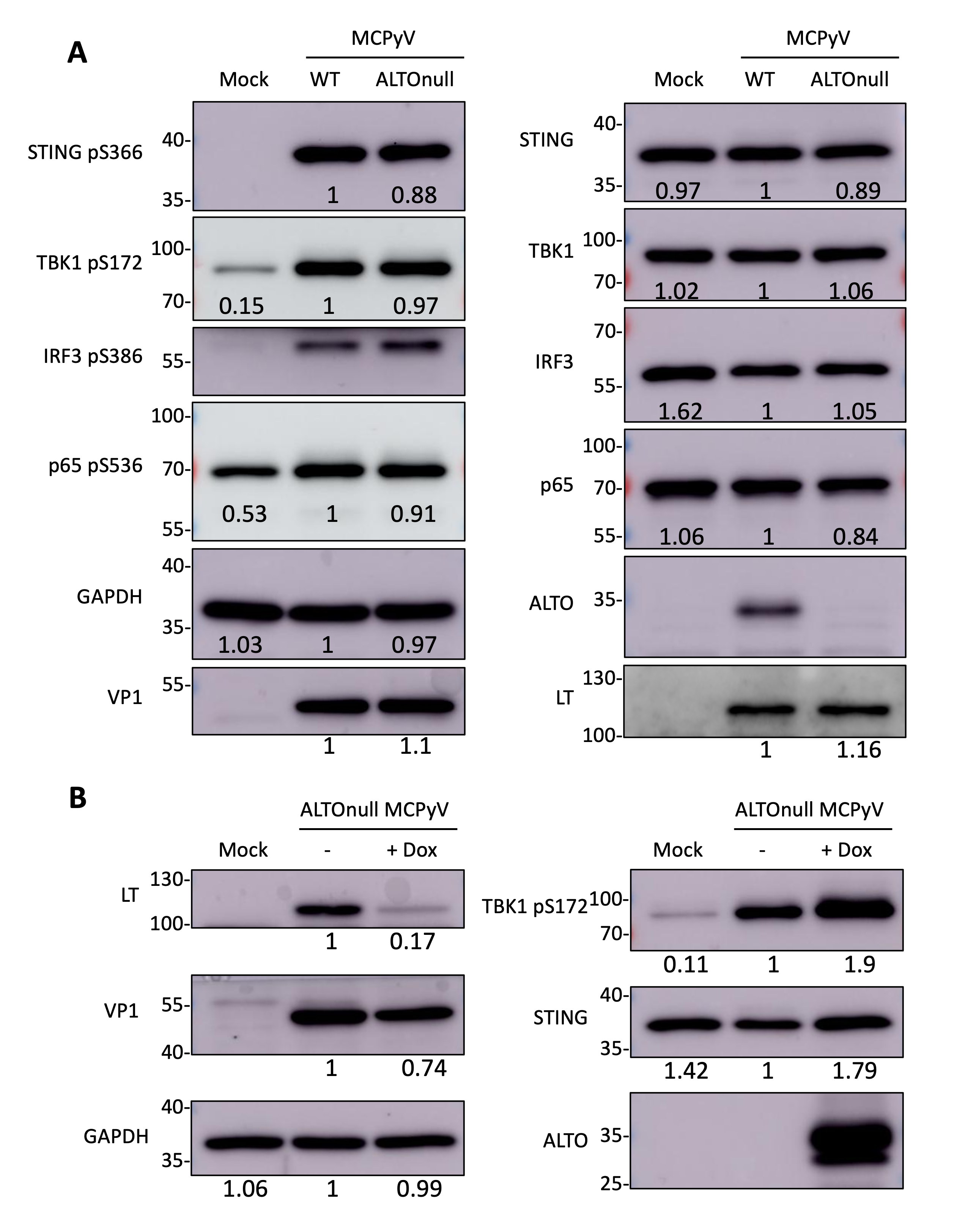

Supplement: S14 Fig — A. Normal HDFs were mock-infected or infected with WT or ALTOnull MCPyV. Whole cell lysates were collected on day 5 post-infection, resolved by SDS/PAGE, and immunoblotted with the indicated antibodies. Quantifications were performed in ImageJ by setting the value of the WT infection to 1. B. HDF-inALTO cells were infected with ALTOnull MCPyV. Cells were either mock-treated or induced with Dox on days 2 and 4 post-infection. Whole cell lysates were collected on day 5 post-infection, resolved by SDS/PAGE, and immunoblotted with the indicated antibodies. Quantifications were performed in ImageJ by setting the value of the no-Dox infection control to 1. (TIF) [file ppat.1012170.s014.tif]

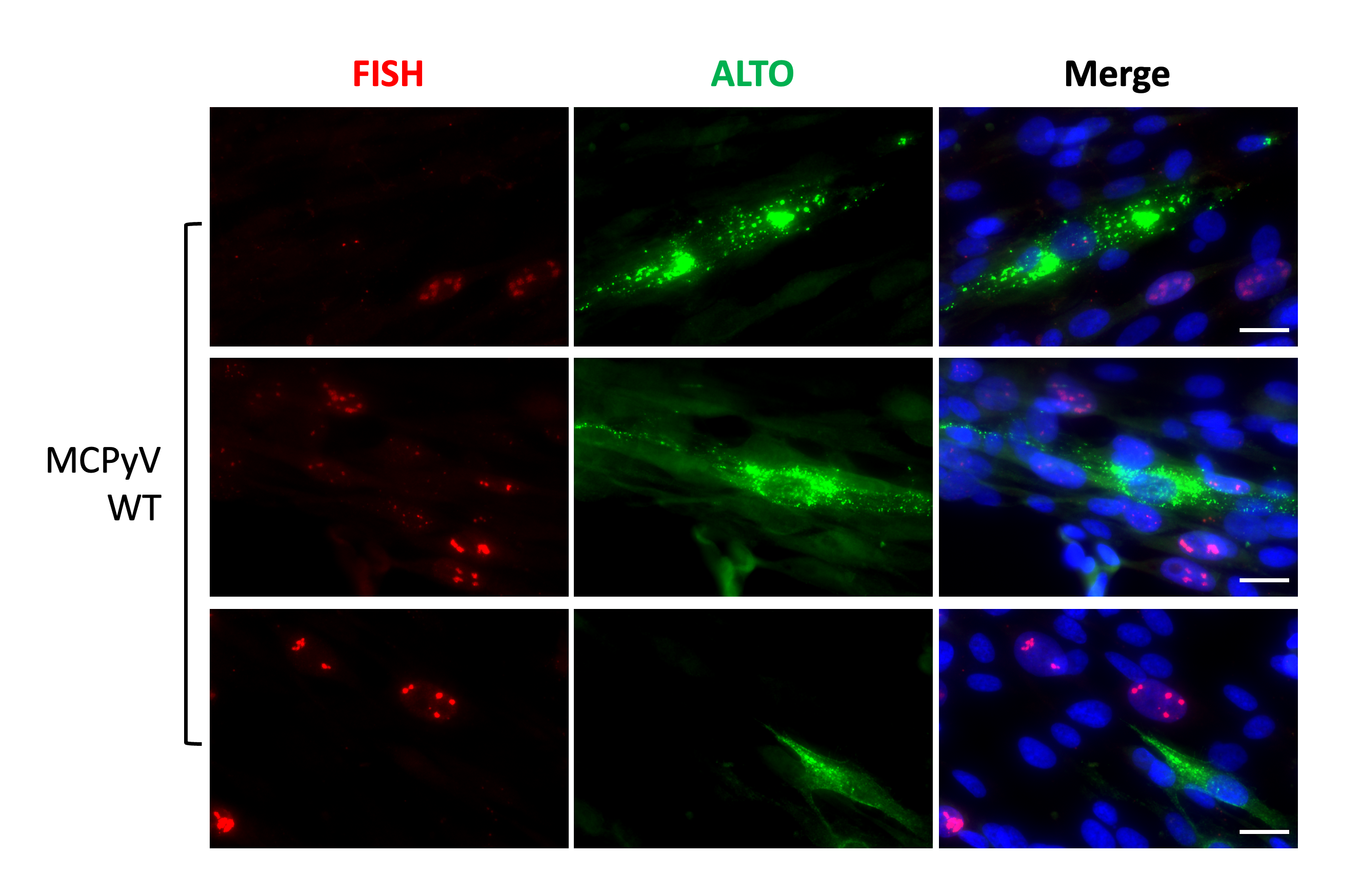

Supplement: S15 Fig — Normal HDFs were mock-infected or infected with 6x10^8^ copies of WT or ALTOnull MCPyV per 48-well. On day 5 post-infection, cells were fixed and immunostained for ALTO, then subjected to FISH using an MCPyV probe, and finally counterstained with DAPI. Scale bar, 20 μm. (TIF) [file ppat.1012170.s015.tif]
